# *Neospora caninum* infection in aborting bovines and lost fetuses: A systematic review and meta-analysis

**DOI:** 10.1371/journal.pone.0268903

**Published:** 2022-05-23

**Authors:** Tooran Nayeri, Mahmood Moosazadeh, Shahabeddin Sarvi, Ahmad Daryani

**Affiliations:** 1 Toxoplasmosis Research Center, Mazandaran University of Medical Sciences, Sari, Iran; 2 Department of Parasitology, School of Medicine, Mazandaran University of Medical Sciences, Sari, Iran; 3 Student Research Committee, Mazandaran University of Medical Sciences, Sari, Iran; 4 Gastrointestinal Cancer Research Center, Non-communicable Diseases Institute, Mazandaran University of Medical Sciences, Sari, Iran; Agricultural Research Service, UNITED STATES

## Abstract

**Background:**

*Neospora caninum* (*N*. *caninum*) is known to be a major cause of reproductive failure in cattle herds around the world. Therefore, the current comprehensive study was performed to estimate the global prevalence of *N*. *caninum* infection in bovines that had an abortion and aborted fetuses.

**Methods:**

In this study, PubMed, ScienceDirect, Web of Science, Scopus, and ProQuest databases were systematically searched for relevant studies up until November 4, 2021. Pooled prevalence and corresponding 95% confidence intervals (CI) were estimated using a random effect model. Other analyzes performed on the data of this study include sensitivity analysis, publication bias test, and quality assessment.

**Results:**

The final analyses included 71 studies conducted on 2965 abortive cattle and 4805 aborted fetuses. The overall prevalence rates of *N*. *caninum* infection in bovines that had an abortion were 47% and 1% using serological and molecular methods. Furthermore, overall prevalence rates of *N*. *caninum* infection in bovine aborted fetuses globally were 35% (95% CI: 8%–62%) and 43% (95% CI: 35%–52%) using serological and molecular methods.

**Conclusions:**

The results of this study showed the high prevalence of *N*. *caninum* infection in bovines that had an abortion and aborted fetuses. It is hoped that the results of this study will help prevent abortion in bovines around the world and encourage further studies to determine the impact of this parasite on the occurrence of abortion that may help reduce the economic damage caused by abortion worldwide.

## Introduction

Abortion is the delivery of an immature fetus (alive or dead) before the end of pregnancy, which occurs as a result of the failure of pregnancy control mechanisms [1]. Infectious agents such as bacteria, viruses, fungi, and protozoa can play an important role in abortion. Among protozoa, *Neospora caninum* (*N*. *caninum*) is the most common cause of reproductive failure in bovines [2]. Bovines can become infected horizontally via the ingestion of feed and water contaminated with sporulated oocysts shed by dogs as the definitive hosts or vertically (transplacentally) by the transmission of the parasite from a dam to a fetus, which is considered the main route of infection in cattle [3, 4]. Endogenous transplacental transmission is due to the recrudescence of the infection during pregnancy in a persistently infected dam, whereas, exogenous transplacental transmission occurs after the initial infection of the pregnant dam following the ingestion of sporulated oocysts [5, 6]. Overall, *N*. *caninum* infection in non-pregnant cattle is latent and asymptomatic. Nevertheless, in pregnant cattle, primary infection or recrudescence may lead to abortion, the birth of a weak calf, or the birth of a clinically normal but chronically infected calf [7, 8]. Various factors such as the virulence of *N*. *caninum*, routes of parasite transmission (vertical or horizontal), type of infection (primary infection, recrudescence, and reinfection), immunological competence of the mother, and stage of pregnancy in which the dam is infected can play a key role in determining infection outcome [8]. Abortion is the most important clinical sign of neosporosis and the majority of the cases occur sporadically, endemically, or epidemically in the sixth month of pregnancy. The rate of congenital transmission is 50–95% and plays an important role in keeping the parasite within the herds [9, 10]. Despite extensive studies on *N*. *caninum* infection, the pathogenesis of *N*. *caninum*-induced abortion is complex and still not well understood. Also, *N*. *caninum* is one of the main constraints to the livestock industry that can lead causes to calve loss, possible loss of milk yield, male infertility, as well as costs associated with establishing the diagnosis of the disease [11–13]. Therefore, given that abortion in bovines is a serious problem and causes significant economic losses to the dairy industry around the world, the main objective of this study was to provide data about the prevalence of *N*. *caninum* infection in bovines that had an abortion and aborted fetuses by molecular, serological, immunohistochemical (IHC), and histopathological methods worldwide.

## Methods

### Study design and protocol registration

This extensive research was reported in accordance with the items reported in the Preferred Reporting Items for Systematic Reviews and Meta-Analyses guidelines (S1 Checklist) [14]. The details of the protocol were registered in PROSPERO with the registration number CRD42020216694.

### Search strategy

To evaluate the global prevalence of *N*. *caninum* infection in bovines that had an abortion and aborted fetuses, the literature search was conducted for relevant papers in 5 English-language databases (PubMed, ScienceDirect, Web of Science, Scopus, and ProQuest) until November 4, 2021, using a combination of keywords related to (“*Neospora caninum*” OR neosporosis) AND (abortion OR miscarriage OR “reproductive failure” OR “fetal loss”) AND (livestock OR ruminant OR cattle OR bovine OR cow). The references of all the original articles in this study were reviewed so that a relevant article would not be missed. All the retrieved articles were saved in EndNote (version X9) to manage the references.

### Inclusion and exclusion criteria

Studies meeting the following criteria were considered eligible: cross-sectional and short communication studies investigating the prevalence of *N*. *caninum* infection in bovines that had an abortion and aborted fetuses with different diagnostic methods (serological, molecular, IHC, and histopathological), full-text articles available online in English language without limitations regarding publication date. Articles examining the relationship between abortion and *N*. *caninum*, studies examining the prevalence of *N*. *caninum* in bovines with more than one abortion, case-control studies, review articles, systematic review and meta-analysis articles, dissertations, conference papers, book chapters, experimental studies, and papers with unclear result sections were excluded from this systematic review and meta-analysis.

### Study selection and data extraction

The initial records obtained during databases searching were imported directly to Endnote X9 software. Following the removal of duplicates, two trained researchers independently evaluated titles, abstracts, and full texts. In the event of a dispute, another author arbitrated and resolved any disagreements. In the next step, the required information was extracted for each study including the name of the first author, publication year, place of study, type of samples, diagnostic methods, sample size (the number of examined animals), results of serological, molecular, IHC, and histopathological methods (number of positive samples). In order to extract data on bovine aborted fetuses, the number of aborted fetuses was included in the study, not the number of samples that were evaluated from different organs of a fetus. To extract data related to the serum of bovines that had an abortion, maternal serum or serum of dam was included in the study, and in cases where both samples were presented in the study, only maternal serum was included and if in one study, maternal serum for half of the samples and serum of dam for the other half was evaluated, the total results of maternal serum and serum of dam were analyzed together. When more than one diagnostic method was used in articles on aborted cattle, the results of the enzyme-linked immunosorbent assay (ELISA) test were analyzed because most studies used the ELISA method. However, in aborted fetuses, because most studies used the indirect immunofluorescence assay (IFA) method, the results of this test were analyzed.

### Quality assessment

The quality of articles was assessed using the Newcastle-Ottawa Scale (NOS) [15]. This quality scale ranges from 0 to 9 points, and higher scores indicate better quality studies. As a result, articles of acceptable quality (≥3 for each study) were included in this study.

### Statistical analysis

The present meta-analysis was carried out using Stata version 14 (Stata Corp, College Station, TX, USA). Pooled prevalence and 95% confidence intervals (CI) were estimated using the random-effects model. Also, the I-squared test was applied to evaluate the heterogeneity index; I-squared values of lower than 25%, 25–50%, and higher than 50% were considered as low, moderate, and high heterogeneity, respectively [16]. The publication bias was examined by Egger’s test. Furthermore, the current study benefited from sensitivity analyses of articles. In this study, subgroup analysis was conducted based on diagnostic methods.

## Results

### Identification and selection of studies

**Fig 1:**
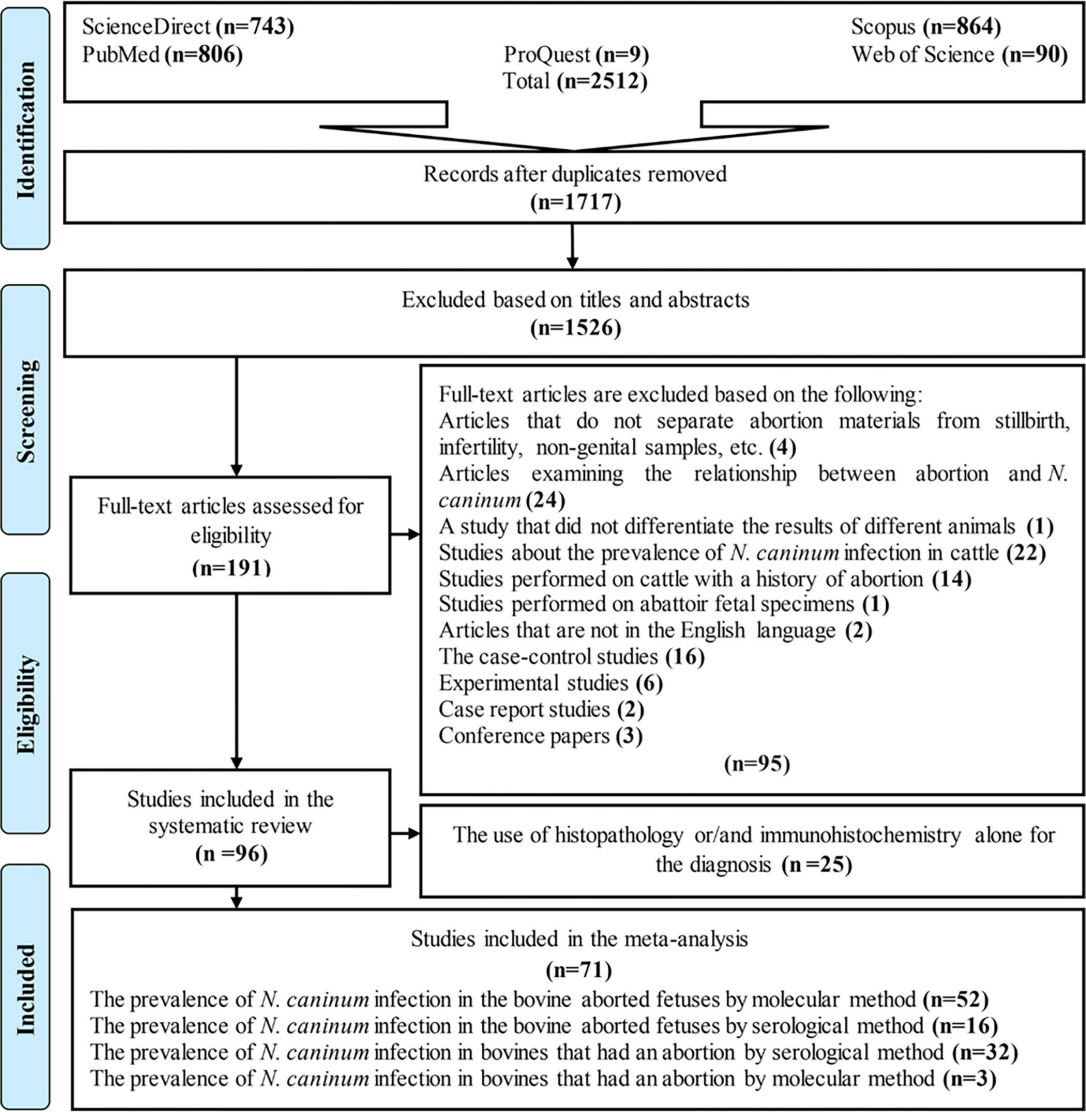
Flow diagram of the study design process.

Our preliminary search of five databases yielded 2512 articles, 1717 articles remained after duplicate removal. Following an initial screening based on titles and abstracts, 1526 studies were excluded. In the next step, the remaining 191 full-text articles were assessed. Finally, 71 of these articles were entered into the meta-analysis with respect to the inclusion/exclusion criteria (Fig 1). Information and characteristics of the investigated articles are presented in Tables 1 and 2.

**Table 1:** Description of the studies included the prevalence of *N*. *caninum* in bovines that had an abortion.

Id	First author (Publication year)	Place of study	Sample	Method	Sample size (n)	Serological results n (%)	Cut off	Molecular results n (%)
1	Reichel and Drake, 1996 [17]	New Zealand	Serum	ELISA and IFA	76	27 (35.52)	1: 200	--
2	Buxton *et al*., 1997 [18]	Scotland	Serum	IFA	465	81 (17.4)	1: 512 ≤	--
3	Campero *et al*., 1998 [19]	Argentina	Serum	IFA	9	8 (88.88)	1: 800	--
4	Cox *et al*., 1998 [20]	New Zealand	Serum	IFA	11	9 (81.81)	--	--
5	Venturini *et al*., 1999 [21]	Argentina	Serum	IFA, NAT, and ELISA	189	122 (64.55)	1: 800	--
6	Pitel *et al*., 2001 [22]	France	Serum	ELISA	163	48 (29.45)	1: 100	--
7	Morales *et al*., 2001 [23]	Mexico	Serum	ELISA	32	29 (90.62)	--	--
8	De Meerschman *et al*., 2002 [24]	Belgium	Serum	IFA	163	33 (20.24)	≥ 1: 25	--
9	Václavek *et al*., 2003 [25]	Czech Republic	Serum	ELISA and IFA	463	18 (3.9)	≥ 1: 640	--
10	Sadrebazzaz *et al*., 2004 [26]	Iran	Serum	IFA	139	27 (19.42)	1: 200	--
11	López‐Gatius *et al*., 2004 [27]	Spain	Serum	ELISA	38	29 (76.31)	--	--
12	Hall *et al*., 2005 [28]	Australia	Serum	ELISA	8	2 (25)	--	--
13	Santos *et al*., 2005 [29]	Brazil	Serum	IFA	35	5 (14.28)	≥ 200	--
14	McInnes *et al*., 2006 [30]	Australia	Serum	IFA, ELISA, and nested-PCR	42	37 (88.10)	--	13 (30.95)
15	Sadrebazzaz *et al*., 2007 [31]	Iran	Serum	IFA	12	6 (50)	1: 200	--
16	Zhang *et al*., 2007 [32]	China	Serum	ELISA	16	12 (75)	--	--
17	Yao *et al*., 2009 [33]	China	Serum	ELISA and nested PCR	20	8 (40)	--	0/20 (0)
18	Basso *et al*., 2010 [34]	Germany	Serum	ELISA	43	38 (88.37)	--	--
19	Nematollahi *et al*., 2011 [35]	Iran	Serum	ELISA and dot-ELISA	32	ELISA: 7 (21.87) and dot-ELISA: 5 (15.62)	--	--
20	Shabbir *et al*., 2011 [36]	Pakistan	Serum	ELISA	141	66 (46.8)	--	--
21	Ghalmi *et al*., 2011 [37]	Algeria	Serum	IFA	5	4 (80)	> 1: 200	--
22	Yang *et al*., 2012 [38]	China	Serum	ELISA	80	28 (35)	--	--
23	Nematollahi *et al*., 2013 [39]	Iran	Serum	ELISA	76	14 (18.42)	--	--
24	Razmi *et al*., 2013 [40]	Iran	Serum	ELISA	200	38 (19)	--	--
25	Şuteu *et al*., 2013 [41]	Romania	Serum	ELISA	9	5 (55.55)	1: 100	--
26	Gavrilović *et al*., 2013 [42]	Serbia	Serum	ELISA	27	7 (25.93)	--	--
27	Gharekhani, 2014 [43]	Iran	Serum	ELISA	85	55 (64.70)	--	--
28	Špilovská *et al*., 2015 [44]	Slovak Republic	Serum	ELISA	4	3 (75)	--	--
29	de Macedo *et al*., 2017 [45]	Brazil	Serum	ELISA and PCR	41	21 (51.2)	1: 100	0 (0)
30	Serrano-Martínez *et al*., 2019 [46]	Peru	Serum	ELISA	219	102 (46.6)	--	--
31	Perotta *et al*., 2021 [47]	Brazil	Serum	No data	73	44 (60.27)	--	--
32	Köse *et al*., 2021 [48]	Turkey	Serum	ELISA	49	8 (16.33)	--	--

ELISA: enzyme-linked immunosorbent assay, IFA: indirect immunofluorescence assay, NAT: *N*. *caninum* agglutination test, PCR: polymerase chain reaction, and Nested-PCR: nested-polymerase chain reaction.

**Table 2:** Characteristics of the included studies for prevalence of *N*. *caninum* in the bovine aborted fetuses.

Id	First author (Publication year)	Place of study	Sample	Methods	Sample size (n)	Serological results n (%)	Molecular results n (%)	Histopathology and IHC results n (%)
1	Thilsted and Dubey, 1989 [49]	USA	Tissue specimens from multiple fetal organs	Histopathology and IHC	9	--	--	Histopathology: 7/9 (77.77) and IHC: 3/9 (33.33)
2	Barr *et al*., 1991 [50]	USA	Brain	IHC	86	--	--	IHC: 72/86 (83.72)
3	Conrad *et al*., 1993a [51]	USA	Brain	Histopathology and IHC	2	--	--	Histopathology: 2/2 (100) and IHC: 2/2 (100)
4	Ogino *et al*., 199) [52]	Japan	Brain	Histopathology and IHC	115	--	--	Histopathology: 3/115 (2.60) and IHC: 2/115 (1.74)
5	Nietfeld *et al*., 1992 [53]	USA	Brain, heart, lung, liver, kidney, placenta, and skeletal muscle	Histopathology and IHC	664	--	--	Histopathology: 25/664 (3.76) and IHC: 21/664 (3.16)
6	Jardine and Last, 1995 [54]	South Africa	Brain and myocardium	Histopathology and IHC	144	--	--	Histopathology: 2/144 (1.39) and IHC: 2/144 (1.39)
7	Obendorf *et al*., 1995 [55]	USA	Brain, heart, kidney, liver, and lung	Histopathology and IHC	11	--	--	Histopathology: 11/11 (100) and IHC: 3/11 (27.27)
8	Jamaluddin *et al*., 1996 [56]	USA	Placenta, fetal tissues, and uterine fluid	Histopathology	595	--	--	Histopathology: 71/595 (11.93)
9	McAllister *et al*., 1996 [57]	USA	Brain	Histopathology and IHC	8	--	--	Histopathology: 8/8 (100) and IHC: 7/8 (90)
10	Buxton *et al*., 2002 [7]	Scotland	Serum	IFA	547	87 (15.9)	--	--
11	Campero *et al*., 1998 [19]	Argentina	Brain, heart, lung, liver, adrenal glands, spleen, kidney, thymus, and skeletal muscle	Histopathology and IHC	2	--	--	Histopathology: 2/2 (100) and IHC: 2/2 (100)
12	Perez *et al*., 1998 [58]	Costa Rica	Tissue	IHC	6	--	--	IHC: 1/6 (16.66)
13	Gottstein *et al*., 1998 [59]	Switzerland	Brain and fetal heart blood or body cavity fluid samples	Histopathology, IFA, ELISA, and PCR	83	7 (8.43)	24 (28.91)	Histopathology: 18/24 (75)
14	Moen *et al*., 1998 [60]	Netherlands	Brain, heart, and liver	Histopathology and IHC	51	--	--	Histopathology: 50/51 (98.03) and IHC: 40/51 (78.43)
15	Hattel *et al*., 1998 [61]	USA	Brain, heart, placenta, kidney, liver, and skeletal muscle	Histopathology	688	--	--	Histopathology: 34/688 (4.94)
16	Baszler *et al*., 1999 [62]	USA	Brain, heart, kidney, liver, lung, spleen, and placenta	Histopathology, IHC, and PCR	61	--	30 (49.18)	Histopathology: 34/61 (55.73) and IHC 26/61 (42.62)
17	Venturini *et al*., 1999 [21]	Argentina	Brain and serum	Histopathology, IFA, agglutination test, and ELISA	104	21 (20.19)	--	Histopathology: 7/8 (87.5)
18	González *et al*., 1999 [63]	Spain	Brain and fetal fluids	Histopathology, IHC, and IFA	81	32/63 (50.79)	--	Histopathology: 36/81 (44.44) and IHC: 25/34 (73.53)
19	Slotved *et al*., 1999 [64]	Denmark	Fetal fluids	Histopathology, IHC, ELISA, and IFA	32	14 (43.75)	--	Histopathology: 14/32 (43.75) and IHC: 14/32 (43.75)
20	Wouda *et al*., 1999 [65]	Netherlands	Brain, heart, and liver	Histopathology	305	--	--	Histopathology: 221/305 (72.46)
21	Atkinson *et al*., 2000 [66]	New South Wales	Fetal tissues	Histopathology	12	--	--	Histopathology: 8/12 (66.66)
22	Pitel *et al*., 2001 [22]	France	Brain	PCR	104	--	22 (21.15)	--
23	Morales *et al*., 2001 [67]	Mexico	Brain, myocardium, diaphragmatic muscle, liver, lung, kidney, and spleen	Histopathology and IHC	211	--	--	Histopathology: 73/211 (34.6) and IHC: 41/53 (77.36)
24	Morales *et al*., 2001 [23]	Mexico	Tissue	Histopathology and IHC	32	--	--	Histopathology: 22/32 (68.75) and IHC: 17/21 (81)
25	Collantes-Fernández *et al*., 2002 [68]	Spain	Brain	Histopathology, real-time PCR, and nested-PCR	12	--	9 (75)	Histopathology: 6/12 (50)
26	Kim *et al*., 2002 [69]	Korea	Brain, heart, lung, liver, spleen, kidney, spinal cord, skeletal muscle, stomach, and small and large intestines	Histopathology, IHC, IFA, and PCR	180	38 (21.11)	34/45 (75.55)	Histopathology: 45/180 (25) and IHC: 38/45 (84.44)
27	Corbellini *et al*., 2002 [70]	Brazil	Brain, heart, lung, liver, kidney, and skeletal muscle	Histopathology and IHC	46	--	--	Histopathology: 22/46 (47.83) and IHC: 18/22 (81.81)
28	De Meerschman *et al*., 2002 [24]	Belgium	Brain, heart, liver, and serum	Histopathology, IHC, and IFA	224	10/166 (6.02)	--	Histopathology: 17/224 (7.59) and IHC: 12/17 (70.59)
29	Campero *et al*., 2003 [71]	Argentina	Brain, heart, lung, liver, adrenal glands, spleen, kidney, thymus, and skeletal muscle	Histopathology and IHC	288	--	--	Histopathology: 43/288 (14.93) and IHC: 26/43 (60.46)
30	Pereira-Bueno *et al*., 2003 [72]	Spain	Brain, heart, and fetal sera or thoracic fluids	Histopathology, IHC, IFA, ELISA, and PCR	80	6/56 (10.7)	9/59 (15.3)	Histopathology: 25/80 (31.3) and IHC: 7/13 (53.8)
31	Boger and Hattel, 2003 [73]	USA	Adrenal gland, brain, heart, intestine, kidney, liver, lung, lymph node, placenta, spleen, skeletal muscle, and thymus	Histopathology and IHC	144	--	--	Histopathology: 65/144 (45.14) and IHC: 12/144 (8.33)
32	Kashiwazaki *et al*., 2004 [74]	Uruguay	Brain	IHC	2	--	--	IHC: 2/2 (100)
33	López‐Gatius *et al*., 2004 [27]	Spain	Brain	Histopathology, IHC, and PCR	2	--	2 (100)	Histopathology: 2/2 (100) and IHC: 2/2 (100)
34	Habibi *et al*., 2005 [75]	Iran	Brain	Semi-nested PCR	6	--	4 (66.66)	--
35	Khodakaram-Tafti and Ikede, 2005 [76]	Canada	Brain and heart	Histopathology and IHC	10	--	--	Histopathology: 5/10 (50) and IHC: 5/10 (50)
36	Hall *et al*., 2005 [28]	Australia	Placenta	Histopathology	7	--	--	Histopathology: 1/7 (14.28)
37	Santos *et al*., 2005 [29]	Brazil	Fetal tissues	IHC	5	--	--	IHC: 5/5 (100)
38	Collantes-Fernández *et al*., 2006 [77]	Spain	Brain, heart, liver, kidney, and lung	Nested-PCR	220	--	72 (32.7)	Histopathology: 18/24 (75)
39	Corbellini *et al*., 2006 [78]	Brazil	Brain and/or muscle (cardiac and skeletal), liver, lung, and kidney	Histopathology and IHC	161	--	--	Histopathology: 37/161 (22.98) and IHC: 34/37 (91.89)
40	McInnes *et al*., 2006 [30]	Australia	Fetal tissues and serum	Histopathology, IFA, ELISA, and nested-PCR	42	--	21/42 (50)	Histopathology: 9/19 (47.36)
41	Medina *et al*., 2006 [79]	Mexico	Brain	Histopathology and nested-PCR	44	--	35 (79.54)	Histopathology: 20/44 (45.45)
42	Razmi *et al*., 2007 [80]	Iran	Brain	Histopathology, IHC, and PCR	100	--	13 (13)	Histopathology: 12/53 (22.64) and IHC: 3/53 (5.66)
43	Reitt *et al*., 2007 [81]	Switzerland	Brain	Real-time PCR and IHC	223	--	36/76 (47.36)	IHC: 4/223 (1.79)
44	Sadrebazzaz *et al*., 2007 [31]	Iran	Fetal sera and fluids and brain	Histopathology, IFA, and semi nested-PCR	12	5 (41.66)	4 (33)	Histopathology: 3/12 (25)
45	Zhang *et al*., 2007 [32]	China	Brain, liver, kidney, heart, lung, muscle, and spleen	Histology, IHC, and PCR	12	--	4 (33.33)	Histopathology: 1/2 (50) and IHC: 1/2 (50)
46	Pabón *et al*., 2007 [82]	Spain	Brain	Histopathology and PCR	7	--	6 (85.71)	Histopathology: 6/7 (85.71)
47	Pescador *et al*., 2007 [83]	Brazil	Brain, heart, lung, liver, kidney, and skeletal muscle	Histopathology and IHC	258	--	--	Histopathology: 89/258 (34.49) and IHC: 55/258 (21.31)
48	Escamilla *et al*., 2007 [84]	Mexico	Lung, myocardium, liver, and kidney	Histopathology	16	--	--	Histopathology: 10/16 (62.5)
49	Moore *et al*., 2008 [85]	Argentina	Fetal fluids, brain, heart, liver, muscle, and placenta	Histopathology, IHC, IFA, and nested-PCR	666	31/55 (56.4)	34/70 (48.5)	Histopathology: 70/666 (10.5) and IHC: 49/70 (70)
50	Yao *et al*., 2009 [33]	China	Brain, heart, lung, liver, spleen, kidney, and skeletal muscle	Nested PCR	26	--	15 (57.7)	--
51	Yildiz *et al*., 2009 [86]	Turkey	Heart, liver, lung, brain, and lymph nodes	Histopathology and IHC	55	--	--	Histopathology: 6/55 (10.90) and IHC: --
52	Salehi *et al*., 2009 [87]	Iran	Brain and placenta	Histopathology and nested-PCR	19	--	17 (89.47)	Histopathology: 19/19 (100)
53	Sánchez *et al*., 2009 [88]	Mexico	Brain	Histopathology, IHC, and PCR	48	--	NC5: 12/29 (41.37) and ITS1: 15/29 (51.72)	Histopathology: 29/48 (60.41) and IHC: 21/29 (72.41)
54	Cabral *et al*., 2009 [89]	Brazil	Brain, heart, kidney, liver, lung, spleen, thymus, and placenta	Histopathology, IHC, and nested-PCR	105	--	23 (21.90)	Histopathology: 75/105 (71.43) and IHC: 9/105 (8.6)
55	Razmi *et al*., 2010 [90]	Iran	Brain and fetal fluids	IHC, ELISA, and PCR	151	15 (9.93)	18 (11.92)	IHC: 6/52 (11.54)
56	Basso *et al*., 2010 [34]	Germany	Brain	PCR	20	--	18 (90)	--
57	Suteu *et al*., 2010 [91]	Romania	Brain and heart	Histopathology and PCR	9	--	3 (33.33)	Histopathology: 0/9 (0)
58	Ghalmi *et al*., 2011 [37]	Algeria	Brain	Histopathology, PCR, and real-time PCR	5	--	3 (60)	Histopathology: 1/5 (20)
59	Tramuta *et al*., 2011 [92]	Italy	Abomasal content, brain, lung, spleen, liver, kidney, and muscle	Multiplex PCR	50	--	7 (14)	--
60	dos Santos DS, 2011 [93]	Brazil	Central nervous system, heart, skeletal muscle, liver, lung, kidney, spleen, thymus, lymph nodes, ovary, testicle, uterus, and ear skin	Histopathology, IHC, and PCR	24	--	5 (20.83)	Histopathology: 8/24 (33.33) and IHC: 3/24 (12.5)
61	Yang *et al*., 2012 [38]	China	Brain	Nested-PCR	80	--	25 (31.3)	--
62	Suteu *et al*., 2012 [94]	Romania	Brain and heart	PCR	21	--	8 (38.09)	--
63	Nematollahi *et al*., 2013 [39]	Iran	Brain, spinal cord, placenta, liver, and heart	Histopathology and PCR	14	--	6 (42.86)	Histopathology: 14/14 (100)
64	Razmi *et al*., 2013 [40]	Iran	Brain	PCR	200	--	23 (11.5)	--
65	Suteu *et al*., 2013 [41]	Romania	Brain and heart	Histopathology, IHC, and PCR	9	--	4 (44.44)	Histopathology: 9/9 (100) and IHC: 2/9 (22.22)
66	Kamali *et al*., 2014 [95]	Iran	Brain	Histopathology and PCR	395	--	179 (45.31)	Histopathology: 16/56 (28.57)
67	Spilovska *et al*., 2015 [44]	Slovak Republic	Brain and serum	ELISA and PCR	4	3 (75)	3 (75)	--
68	Salehi *et al*., 2015 [96]	Iran	Brain	Nested-PCR	16	--	12 (75)	--
69	Medina-Esparza *et al*., 2016 [97]	Mexico	Brain	Nested-PCR	63	--	27 (42.86)	--
70	Ozkaraca *et al*., 2017 [98]	Turkey	Brain, myocardium, liver, lung, kidney, spleen, and thymus	IHC and Duplex PCR	102	--	26 (25.49)	IHC: 18/102 (17.64)
71	de Macedo *et al*., 2017 [45]	Brazil	Blood, intrathoracic fluid, brain, heart, liver, and lung	Histopathology, IHC, ELISA, and PCR	41	8/30 (26.7)	14/36 (38.8)	Histopathology: 29/36 (80.55) and IHC: 9/36 (25)
72	Kaveh *et al*., 2017 [99]	Iran	Brain, kidney, spleen, liver, and lung	PCR and RT-PCR	128	--	39 (30.47)	--
73	Qian *et al*., 2017 [100]	China	Brain, heart, lung, liver, spleen, kidney, and skeletal muscle	Nested-PCR	7	--	4 (57.14)	--
74	Diaz Cao *et al*., 2018 [101]	Spain	Brain	Real-time PCR	25	--	2 (8)	--
75	Tian *et al*., 2018 [102]	China	Fetal tissues	LF-RPA and nested-PCR	75	--	LF-RPA: 18 (24) and nested PCR: 17 (22.6)	--
76	Snak *et al*., 2018 [103]	Brazil	Fetal tissues	PCR	17	--	9 (52.94)	--
77	Moroni *et al*., 2018 [104]	Chile	Brain and optic nerve	Histopathology, IHC, and PCR	296	--	31 (10.5)	Histopathology: 44/296 (14.9) and IHC: 27/44 (61.36)
78	Bartley *et al*., 2019 [105]	Scotland	Brain, heart, and placenta	Nested-PCR	455	--	82 (18.02)	--
79	Acici *et al*., 2019 [106]	Turkey	Brain, spleen, liver, lung, amniotic fluid, and fetal membranes	Real-time PCR	88	--	43 (48.9)	--
80	Mahajan *et al*., 2020 [107]	India	Heart, liver, and brain	Histopathology and IHC	13	--	--	Histopathology: 1/13 (7.69) and IHC: 1/13 (7.69)
81	Amouei *et al*., 2019 [108]	Iran	Brain	Nested-PCR	9	--	2 (22.2)	--
82	Serrano-Martínez *et al*., 2019 [46]	Peru	Fetal tissues and serum	Histopathology, ELISA, and nested-PCR	68	10 (14.70)	11 (16.17)	Histopathology: 5/68 (7.35)
83	Villa *et al*., 2021 [109]	Italy	Brain, lung, and liver	Real-time quantitative PCR	198	--	55 (27.8)	--
84	Salehi *et al*., 2021 [110]	Iran	Brain	Nested-PCR	78	--	16 (20.5)	--
85	Perotta *et al*., 2021 [47]	Brazil	Serum, peritoneal and pleural fluids, brain, heart, lung, liver, spleen, thymus, kidney, and skeletal muscle	Histopathology, IFA, and nested-PCR	5	5 (100)	1/1 (100)	Histopathology: 1/1 (100)
86	Dorsch *et al*., 2021 [111]	Argentina	Thoracic-abdominal fluids, brain, cerebellum, spinal cord, heart, lungs, thymus, tongue, skeletal muscle, spleen, abomasum, intestine, liver, kidney, and adrenal glands	Histopathology, IHC, IFA, and nested-PCR	758	59/99 (59.6)	96/106 (90.6)	Histopathology: 107/758 (14.12) and IHC: 30/62 (48.39)
87	El-Alfy *et al*., 2021 [112]	Japan	Brain	Nested-PCR	5	--	5 (100)	--

IHC: immunohistochemistry, IFA: indirect immunofluorescence assay, ELISA: enzyme-linked immunosorbent assay, PCR: polymerase chain reaction, Real-time PCR: real-time polymerase chain reaction, Nested-PCR: nested-polymerase chain reaction, RT-PCR: reverse transcription polymerase chain reaction, and LF-RPA assay: lateral flow strips- recombinase polymerase amplification.

### General characteristics of the included studies

The publication date of the studied articles was from 1989 to 2021, and all articles were cross-sectional and short communication studies. Overall, there were 26 studies (Spain = 7, Romania = 3, Switzerland = 2, Netherlands = 2, Scotland = 2, Italy = 2, Denmark = 1, France = 1, New South Wales = 1, Serbia = 1, Czech Republic = 1, Belgium = 1, Germany = 1, and Slovak Republic = 1) in Europe, 29 studies (Iran = 15, China = 5, Turkey = 4, Japan = 2, India = 1, Pakistan = 1, and Korea = 1) in Asia, 2 studies (South Africa = 1 and Algeria = 1) in Africa, 35 studies (USA = 10, Brazil = 9, Mexico = 6, Argentina = 5, Costa Rica = 1, Uruguay = 1, Canada = 1, Chile = 1, and Peru = 1) in America and 4 studies (New Zealand = 2 and Australia = 2) in Australia/Oceania. The most common diagnostic tests of serology and molecular utilized in the studies to examine the serum samples of bovines that had an abortion and serum or brain samples of aborted fetuses were the ELISA and polymerase chain reaction (PCR). Some studies have used more than one diagnostic method for *N*. *caninum* infection (Tables 1 and 2).

In addition, the quality assessment of studies with the NOS checklist showed that the articles included in this meta-analysis are of acceptable quality. S1 Table shows the quality scores of various eligible studies.

### Prevalence of *N*. *caninum* infection in bovines that had an abortion

**Fig 2:**
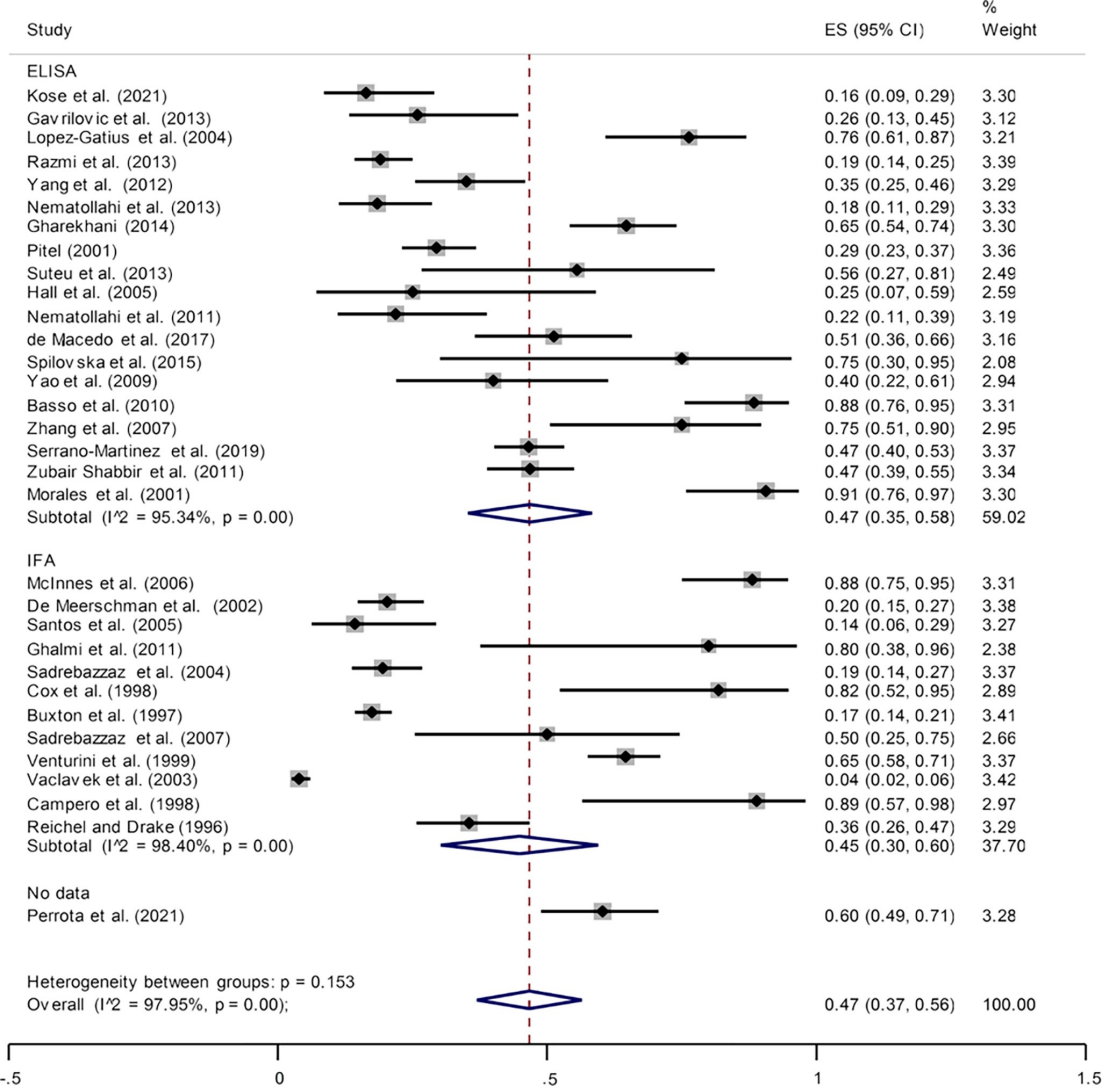
The reported seroprevalence rate of anti- *N*. *caninum* antibodies in bovines that had an abortion by serological methods.

A total of 2965 and 103 bovines that had an abortion were evaluated for the prevalence of *N*. *caninum*, out of which 941 and 13 cases were positive using serological and molecular methods in different geographical locations worldwide. The results indicated that the rate of prevalence of *N*. *caninum* infection was 47% (95% CI: 37%–56%) and 1% (95% CI: -1%–3%) using serological and molecular methods. Heterogeneity were significant in different studies (I^2^ = 89.35%, *p* = 0.000 and I^2^ = 97.95%, *p* = 0.000) (Fig 2 and S1 Fig). Egger’s regression test showed that publication bias exerted a significant influence on the prevalence of *N*. *caninum* infection in bovines that had an abortion (*p* = 0.001) (S2 Fig). The pooled prevalence rates of *N*. *caninum* infection in bovines that had an abortion according to the diagnostic methods of ELISA and IFA were determined to be 47% (95% CI: 35%–58%) and 45% (95% CI: 30%–60%), respectively. One study did not mention the type of serology test and the prevalence was 60% (95% CI: 49%–71%) [47]. The results of the subgroup analysis revealed that the effect of assessment of the detection methods on the heterogeneity of studies was not statistically significant (*p* = 0.533). The results of the sensitivity analysis test showed no significant effect of deleting an article with overall results (S3 Fig).

### Prevalence of *N*. *caninum* infection in bovine aborted fetuses

**Fig 3:**
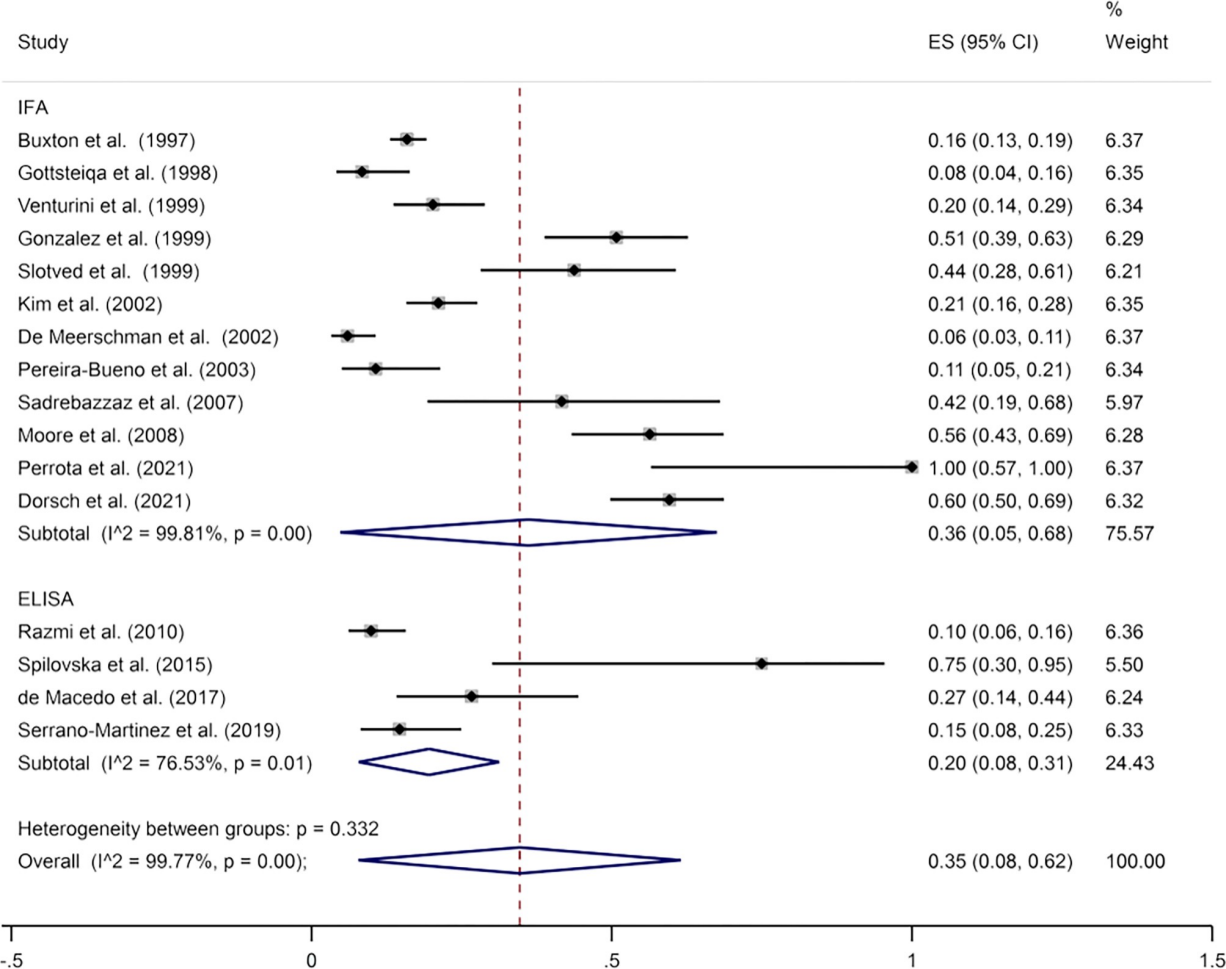
The pooled seroprevalence rate of anti- *N*. *caninum* antibodies in the bovine aborted fetuses.

Among databases searched, a total of 1655 bovine aborted fetuses were examined for the seroprevalence rate of the antibodies against *N*. *caninum*, out of which 351 cases were seropositive using several serological methods. The overall seroprevalence of the antibodies against *N*. *caninum* in bovine aborted fetuses based on the random effect model was calculated at 35% (95% CI: 8%–62%). I-squared statistics showed a high heterogeneity among the studies (I^2^ = 99.77%, *p* = 0.000) (Fig 3). Egger’s test was used to determine the publication bias and the results showed no publication bias on the overall prevalence estimate (*p* = 0.125) (S4 Fig). Based on the meta-analysis, the prevalence of *N*. *caninum* infection in the bovine aborted fetuses based on the diagnostic methods of IFA and ELISA was estimated to be 36% (95% CI: 5%-68%) and 20% (95% CI: 8%− 31%), respectively. The results of the subgroup analysis showed that the effect of diagnostic methods on the heterogeneity of studies was not statistically significant (*p* = 0.595). In addition, the results of the sensitivity analysis showed that the overall estimate did not change with the removal of each study (S5 Fig).

**Fig 4:**
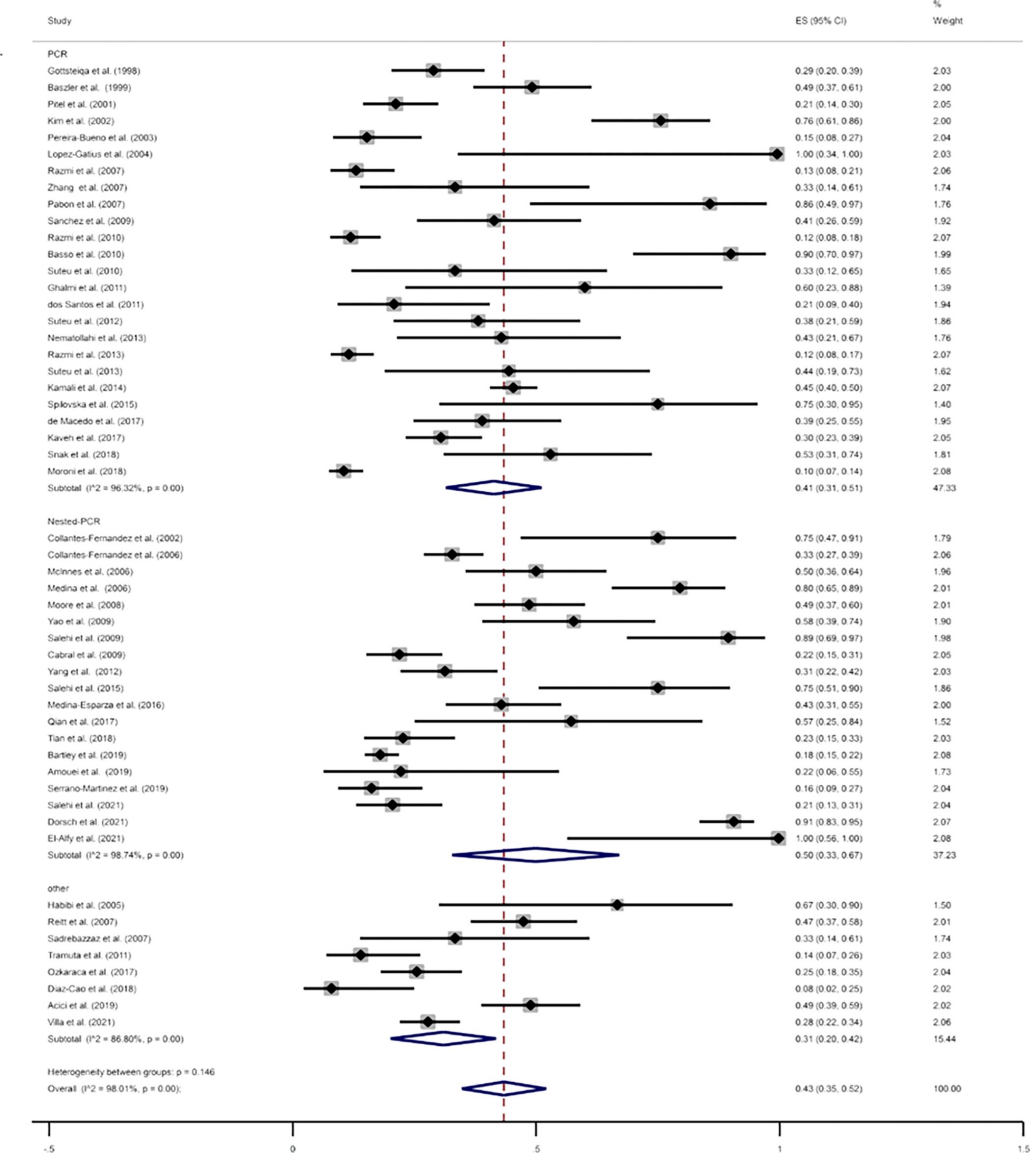
The prevalence of *N*. *caninum* infection in the bovine aborted fetuses using molecular methods.

A total number of 52 eligible studies examined 3888 samples from bovine aborted fetuses, out of which 1219 cases were positive using molecular methods. The global pooled prevalence of *N*. *caninum* infection in bovine aborted fetuses using molecular methods was estimated at 43% (95% CI: 35%–52%) (I^2^ = 98.01%, *p* = 0.00) (Fig 4). The publication bias was significant based on the results of Egger’s test (*p* = 0.000) using molecular methods (S6 Fig). Based on the meta-analysis, the prevalence of *N*. *caninum* infection in the bovine aborted fetuses based on the diagnostic methods of PCR, nested PCR, and others was estimated to be 41% (95% CI: 31%-51%), 50% (95% CI: 33%–67%), and 31% (95% CI: 20%− 42%), respectively. Results of subgroup analysis based on diagnostic methods indicated that the effect of diagnostic methods on the heterogeneity of studies was not statistically significant (*p* = 0.336). In the sensitivity analysis test, the effect of omission of each study on the overall result of the study was evaluated. The findings of this test indicated the stability of the results of the study. In addition, in three articles, 6826 and 2721 samples were examined by histopathology and IHC methods; 1518 and 674 cases were positive for *N*. *caninum* (22.24% and 24.77% positive for neosporosis) (Table 2).

## Discussion

*N*. *caninum* was identified as the main cause of abortion in cattle [49], which is one of the most important economic diseases. Hence, in this systematic review and meta-analysis study, the prevalence of *N*. *caninum* infection in bovines that had an abortion and aborted fetuses was investigated by molecular, serological, IHC, and histopathological methods. Diagnosis of *N*. *caninum* abortion may be inconclusive for the following reasons: 1) expensive and sometimes difficult to diagnose, 2) lack of access to fetus and placenta, especially for beef cattle, and 3) using the serology method alone [45]. Identification of compatible histological lesions, detection of parasites in fetal tissues by PCR or IHC, and detection of specific antibodies in fetal fluids and maternal serum are the diagnostic criteria for *N*. *caninum*–induced abortion [2].

In this systematic review and meta-analysis study, 57 papers performed the histopathological evaluations based on observation of characteristic or compatible lesions with *N*. *caninum* infection, and no analysis was performed on them. According to the results of the included articles, the prevalence of *N*. *caninum* infection in bovine aborted fetuses by histopathology was 22.24%. Since many factors can play a role in abortion, determining the cause is often difficult. Abortions usually show no gross lesions or clinical signs in the fetus, and a history of abortion rarely provides convincing clues to the cause [38]. However, histopathological examination of the aborted fetus and isolation or culture of pathogens are common methods for routine diagnostic examination of materials submitted [38]. The cell culture system is laborious, time-consuming, and relatively low sensitive [38]. Histopathological examination of the fetus is essential for a definitive diagnosis. Nevertheless, histological examinations of tissues from autolyzed fetuses are not possible [113]. Ideally, the entire fetus should be sent, but if this is not possible, samples from the brain, heart, and liver should be examined for histopathological changes and body fluids or serum for serological evaluation. The fetal brain is more damaged than other organs, but the heart and liver are also commonly affected [3]. Focal encephalitis is the most significant lesion that is associated with necrosis and nonsuppurative inflammation particularly, especially in the brain and to a lesser extent in the cord [3]. As the lesion progresses, necrotic areas may be replaced by macrophages, and the glial cells that cause the lesion appear as discrete granuloma [114]. In addition, other techniques, such as IHC, are used to show parasites associated with lesions in aborted fetal tissues. IHC is a relatively insensitive technique for detecting the parasite in host tissues due to the low quality of the fetal tissue (autolyzed, mummified, or macerated) and low parasite numbers that may lead to false negatives [62, 115]. In this study, 2721 samples were evaluated for the presence of *N*. *caninum* in fetal tissues, of which 674 were positive (24.77% positive for neosporosis) by the IHC method. Serology is another method used to reliably diagnose *N*. *caninum*-related abortion problems, but it alone is not enough [39]. Tests such as IFA and ELISA are used for serological diagnosis of neosporosis. IFA is the gold standard for the serological diagnosis of *N*. *caninum* infection and is highly specific. Despite numerous common antigens, there is no evidence of cross-reaction between *N*. *caninum* and *T*. *gondii* [116]. However, indirect ELISA indicates the possibility of cross-reactivity between the sera of animals infected with *N*. *caninum*, *T*. *gondii*, or *Sarcocystis* species and leads to false-positive results [116]. Positive results of serological tests indicate infection of the animal with *N*. *caninum*, but in the case of abortion, serological tests cannot provide a definitive diagnosis. To confirm the diagnosis, fetal tissues should be examined for the presence of specific lesions, tissue cysts, and tachyzoites [117]. Overall, this meta-analysis demonstrated that seroprevalence of *N*. *caninum* infection is 35% in the aborted fetuses of cattle using serological tests. Also, the prevalence of *N*. *caninum* infection in the bovine aborted fetuses using different molecular tests was obtained at 43%. The use of molecular techniques, such as PCR, is useful for the diagnosis of neosporosis in bovines. PCR is a very specific and sensitive technique for the detection of small numbers of parasites in tissue and the ability to amplify small amounts of *N*. *caninum* DNA in a larger quantity of tissue [108, 115]. However, DNA detection in aborted fetuses is not sufficient to confirm that *N*. *caninum* is responsible for reproductive failure because other abortifacient factors may also play a potential role in abortion [106]. Although PCR is one of the most accurate and widely used molecular methods to study the global prevalence of *N*. *caninum* infection in aborting bovines and lost fetuses, it is best to use PCR and IHC tests simultaneously to increase the success of the definitive diagnosis of neosporosis.

In this study, the pooled prevalence rate of *N*. *caninum* infection in bovines that had an abortion was 47% and 1% by serological and molecular methods. Given that the seroprevalence of *N*. *caninum* in cattle is high and the cattle that abort the infected fetus is probably seropositive. Therefore, the maternal serological examination is useful to rule out *N*. *caninum*-associated abortion [36].

*N*. *caninum* causes heavy economic losses in livestock, particularly cattle, which are economically the most important host of natural *N*. *caninum* infections [105]. One of the major effects of infection in cows is abortion, in some geographical areas up to 42.5% of abortions are caused by *N*. *caninum*. In general, the economic impact of neosporosis has several aspects, including losses directly caused by the disease, the costs related to disease prevention, and the value of fetuses lost. The main output of a herd is its products, such as calf, milk, and meat. Indirect costs include costs such as professional help, re-breeding of cows, increased lactation time, decreased production of milk and dairy products, and early replacement of infected animals [11, 118]. In one study, costs of the disease in the New Zealand beef industry were estimated at an average of US $1.1 million due to abortion or infection and in the US, it is estimated that neosporosis costs the dairy industry US $546.3 million annually [119].

In this systematic study, heterogeneity was significant (I^2^ > 50). Geographical factors of each region, differences in the ages of the animals in the different studies, differences in sampling, the study of various tissues to estimate the prevalence in the included studies, and a variety of detection methods can be reasons for high heterogeneity. The lack of evaluation of various associated factors in the eligible studies can be considered a basic gap. The number of bovine aborted fetuses sent to the laboratory was relatively small in some studies, which may limit the ability of the results to generalize. Also, this small number can lead to wide confidence intervals. Another limitation is that this study used only articles published in English language, and articles related to other languages were excluded and this can be one of the reasons for publication bias. To the best of our knowledge, this is the first review that systematically assesses the studies on the prevalence of *N*. *caninum* infection in bovines that had an abortion and aborted fetuses. The results of the meta-analysis demonstrated a high prevalence of neosporosis in bovines that had an abortion and aborted fetuses throughout the world. According to the study, *N*. *caninum* infection could be considered a potential risk factor for reproductive failure in bovines worldwide. These findings provide a better picture of the epidemiology of *N*. *caninum* among bovines that had an abortion and aborted fetuses and may be useful for improving prevention and control strategies in the future as well as helping to reduce significant economic losses to the livestock industry.

## Supporting information

S1 Checklist PRISMA 2009 checklist. (DOC)

S1 Table NOS checklist. (DOCX)

S1 Fig The pooled prevalence of *N*. *caninum* infection in bovines that had an abortion using molecular methods. (DOCX)

S2 Fig Funnel plot to detect publication bias in studies showing the seroprevalence of *N*. *caninum* infection in bovines that had an abortion. (DOCX)

S3 Fig Sensitivity analysis for assessing the effect of each primary study on the total estimates in studies showing the seroprevalence of *N*. *caninum* infection in bovines that had an abortion. (DOCX)

S4 Fig Funnel plot to detect publication bias in studies showing the seroprevalence of *N*. *caninum* infection in the bovine aborted fetuses. (DOCX)

S5 Fig Sensitivity analysis for assessing the effect of each primary study on the total estimates in studies showing the seroprevalence of anti- *N*. *caninum* antibodies in the bovine aborted fetuses. (DOCX)

S6 Fig Funnel plot to detect publication bias in studies showing the prevalence of *N*. *caninum* infection in the bovine aborted fetuses by molecular methods. (DOCX)
