# Supplementary material for: Neospora caninum infection in aborting bovines and lost fetuses: A systematic review and meta-analysis
Source: PLoS One. 2022 May 23;17(5):e0268903. doi: 10.1371/journal.pone.0268903 (PMC9126370; doi:10.1371/journal.pone.0268903)
Supplement: S1 Table — (DOCX) [file pone.0268903.s002.docx]

**Supplementary Table 2**

Quality assessment of included cross-sectional studies based on the Newcastle-Ottawa Scale.

| Id | First author | Publication year | Place of study | Selection (3 points) | Comparability (2 points) | Outcome (3 points) | Total (scores) |
| --- | --- | --- | --- | --- | --- | --- | --- |
| 1 | Thilsted and dubey | 1989 | USA | 2 | 0 | 1 | 3 |
| 2 | Barr *et al*. | 1991 | USA | 2 | 0 | 1 | 3 |
| 3 | Conrad *et al*. | 1993 | USA | 2 | 0 | 1 | 3 |
| 4 | Ogino *et al*. | 1992 | Japan | 2 | 0 | 1 | 3 |
| 5 | Nietfeld *et al*. | 1992 | USA | 2 | 0 | 1 | 3 |
| 6 | Reichel and Drake | 1992 | New Zealand | 3 | 0 | 2 | 5 |
| 7 | Jardine and Last | 1995 | South Africa | 2 | 0 | 1 | 3 |
| 8 | Obendorf *et al*. | 1995 | USA | 2 | 0 | 1 | 3 |
| 9 | Jamaluddin | 1996 | USA | 2 | 0 | 2 | 4 |
| 10 | McAllister *et al*. | 1996 | USA | 3 | 0 | 2 | 5 |
| 11 | Buxton *et al*. | 1997 | Scotland | 2 | 0 | 1 | 3 |
| 12 | Campero *et al*. | 1998 | Argentina | 2 | 0 | 1 | 3 |
| 13 | Perez *et al*. | 1998 | Costa Rica | 2 | 0 | 2 | 4 |
| 14 | Gottstein *et al*. | 1998 | Switzerland | 2 | 0 | 2 | 4 |
| 15 | Moen *et al*. | 1998 | Netherlands | 2 | 0 | 2 | 4 |
| 16 | Hattel *et al*. | 1998 | USA | 2 | 0 | 1 | 3 |
| 17 | Cox *et al*. | 1998 | New Zealand | 2 | 0 | 1 | 3 |
| 18 | Baszler *et al*. | 1999 | USA | 2 | 0 | 1 | 3 |
| 19 | Venturini *et al*. | 1999 | Argentina | 2 | 0 | 1 | 3 |
| 20 | Gonzalez *et al*. | 1999 | Spain | 2 | 0 | 1 | 3 |
| 21 | Slotved *et al*. | 1999 | Denmark | 2 | 0 | 2 | 4 |
| 22 | Wouda et al. | 1999 | Netherlands | 2 | 0 | 2 | 4 |
| 23 | Atkinson *et al*. | 2000 | New South Wales | 2 | 0 | 2 | 4 |
| 24 | Pitel *et al*. | 2001 | France | 2 | 1 | 2 | 5 |
| 25 | Morales *et al*. | 2001 | Mexico | 2 | 0 | 1 | 3 |
| 26 | Morales *et al*. | 2001 | Mexico | 3 | 0 | 1 | 4 |
| 27 | Collantes-Fernandez *et al*. | 2002 | Spain | 2 | 0 | 2 | 4 |
| 28 | Kim *et al*. | 2002 | Korea | 2 | 0 | 1 | 3 |
| 29 | Corbellini *et al*. | 2002 | Brazil | 2 | 0 | 2 | 4 |
| 30 | De Meerschman *et al*. | 2002 | Belgium | 2 | 0 | 2 | 4 |
| 31 | Campero *et al*. | 2003 | Argentina | 2 | 0 | 2 | 4 |
| 32 | Pereira-Bueno *et al*. | 2003 | Spain | 2 | 1 | 2 | 5 |
| 33 | Boger *et al*. | 2003 | USA | 2 | 0 | 2 | 4 |
| 34 | Vaclavek et al. | 2003 | Czech Republic | 2 | 0 | 2 | 4 |
| 35 | Kashiwazaki *et al*. | 2004 | Uruguay | 3 | 0 | 2 | 5 |
| 36 | Lopez-Gatius *et al*. | 2004 | Spain | 3 | 0 | 2 | 5 |
| 37 | Sadrebazzaz *et al*. | 2004 | Iran | 2 | 0 | 2 | 4 |
| 38 | Habibi *et al*. | 2005 | Iran | 2 | 0 | 2 | 4 |
| 39 | Khodakaram-Tafti and Ikede | 2005 | Canada | 2 | 0 | 1 | 3 |
| 40 | Hall *et al*. | 2005 | Australia | 3 | 1 | 2 | 6 |
| 41 | Santos *et al*. | 2005 | Brazil | 2 | 0 | 1 | 3 |
| 42 | Collantes-Fernandez *et al*. | 2006 | Spain | 2 | 0 | 2 | 4 |
| 43 | Corbellini *et al*. | 2006 | Brazil | 2 | 0 | 2 | 4 |
| 44 | McInnes *et al*. | 2006 | Australia | 2 | 0 | 1 | 3 |
| 45 | Medina *et al*. | 2006 | Mexico | 3 | 0 | 2 | 5 |
| 46 | Razmi *et al*. | 2007 | Iran | 2 | 1 | 1 | 4 |
| 47 | Reitt *et al*. | 2007 | Switzerland | 2 | 1 | 1 | 4 |
| 48 | Sadrebazzaz *et al*. | 2007 | Iran | 2 | 0 | 2 | 4 |
| 49 | Zhang *et al*. | 2007 | China | 2 | 0 | 1 | 3 |
| 50 | Pabon *et al*. | 2007 | Spain | 2 | 2 | 2 | 6 |
| 51 | Pescador *et al*. | 2007 | Brazil | 2 | 0 | 2 | 4 |
| 52 | Escamilla *et al*. | 2007 | Mexico | 3 | 1 | 1 | 5 |
| 53 | Moore *et al*. | 2008 | Argentina | 2 | 0 | 2 | 4 |
| 54 | Yao *et al*. | 2009 | China | 2 | 0 | 1 | 3 |
| 55 | Yildiz *et al*. | 2009 | Turkey | 2 | 0 | 2 | 4 |
| 56 | Salehi *et al*. | 2009 | Iran | 3 | 0 | 1 | 4 |
| 57 | Sanchez *et al*. | 2009 | Mexico | 2 | 0 | 2 | 4 |
| 58 | Cabral *et al*. | 2009 | Brazil | 2 | 0 | 1 | 3 |
| 59 | Razmi *et al*. | 2010 | Iran | 3 | 1 | 2 | 6 |
| 60 | Basso *et al*. | 2010 | Germany | 2 | 0 | 1 | 3 |
| 61 | Suteu *et al*. | 2010 | Romania | 2 | 0 | 1 | 3 |
| 62 | Ghalmi *et al*. | 2011 | Algeria | 3 | 0 | 2 | 5 |
| 63 | Tramuta *et al*. | 2011 | Italy | 2 | 0 | 1 | 3 |
| 64 | dos Santos *et al*. | 2011 | Brazil | 2 | 0 | 1 | 3 |
| 65 | Nematollahi *et al*. | 2011 | Iran | 2 | 0 | 2 | 4 |
| 66 | Zubair Shabbir *et al*. | 2011 | Pakistan | 2 | 1 | 2 | 5 |
| 67 | Yang *et al*. | 2012 | China | 2 | 0 | 2 | 4 |
| 68 | Suteu *et al*. | 2012 | Romania | 2 | 1 | 1 | 4 |
| 69 | Nematollahi *et al*. | 2013 | Iran | 2 | 0 | 2 | 4 |
| 70 | Razmi *et al*. | 2013 | Iran | 3 | 0 | 2 | 5 |
| 71 | Suteu *et al*. | 2013 | Romania | 2 | 0 | 1 | 3 |
| 72 | Gavrilovic *et al*. | 2013 | Serbia | 2 | 0 | 2 | 4 |
| 73 | Kamali *et al*. | 2014 | Iran | 2 | 2 | 2 | 6 |
| 74 | Gharekhani | 2014 | Iran | 2 | 2 | 2 | 6 |
| 75 | Spilovska *et al*. | 2015 | Slovak Republic | 3 | 1 | 2 | 6 |
| 76 | Salehi *et al*. | 2015 | Iran | 2 | 0 | 2 | 4 |
| 77 | Medina-Esparza *et al*. | 2016 | Mexico | 2 | 0 | 1 | 3 |
| 78 | Ozkaraca *et al*. | 2017 | Turkey | 2 | 0 | 2 | 4 |
| 79 | de Macedo *et al*. | 2017 | Brazil | 2 | 0 | 2 | 4 |
| 80 | Kaveh *et al*. | 2017 | Iran | 2 | 0 | 1 | 3 |
| 81 | Qian *et al*. | 2017 | China | 3 | 2 | 2 | 7 |
| 82 | Díaz-Cao *et al*. | 2018 | Spain | 2 | 0 | 1 | 3 |
| 83 | Tian *et al*. | 2018 | China | 2 | 0 | 1 | 3 |
| 84 | Snak *et al*. | 2018 | Brazil | 3 | 2 | 2 | 7 |
| 85 | Moroni *et al*. | 2018 | Chile | 2 | 0 | 2 | 4 |
| 86 | Bartley *et al*. | 2019 | Scotland | 2 | 0 | 2 | 4 |
| 87 | Acici *et al*. | 2019 | Turkey | 2 | 0 | 2 | 4 |
| 88 | Mahajan *et al*. | 2019 | India | 2 | 2 | 2 | 6 |
| 89 | Amouei *et al*. | 2019 | Iran | 2 | 0 | 2 | 4 |
| 90 | Serrano-Martinez *et al*. | 2019 | Peru | 2 | 0 | 2 | 4 |
| 91 | Villa *et al*. | 2021 | Italy | 2 | 0 | 2 | 4 |
| 92 | Salehi *et al*. | 2021 | Iran | 2 | 0 | 2 | 4 |
| 93 | Perrota *et al*. | 2021 | Brazil | 3 | 0 | 1 | 4 |
| 94 | Dorsch *et al*. | 2021 | Argentina | 2 | 0 | 2 | 4 |
| 95 | El-Alfy *et al*. | 2021 | Japan | 2 | 0 | 2 | 4 |
| 96 | Kose *et al*. | 2021 | Turkey | 2 | 0 | 2 | 4 |
