# Supplementary material for: Neospora caninum infection in aborting bovines and lost fetuses: A systematic review and meta-analysis
Source: PLoS One. 2022 May 23;17(5):e0268903. doi: 10.1371/journal.pone.0268903 (PMC9126370; doi:10.1371/journal.pone.0268903)
Supplement: S2 Fig — (DOCX) [file pone.0268903.s004.docx]

**Supplementary Fig. 2.** Funnel plot to detect publication bias in studies showing the seroprevalence of *N. caninum* infection in bovines that had an abortion.
