# Supplementary material for: Neospora caninum infection in aborting bovines and lost fetuses: A systematic review and meta-analysis
Source: PLoS One. 2022 May 23;17(5):e0268903. doi: 10.1371/journal.pone.0268903 (PMC9126370; doi:10.1371/journal.pone.0268903)
Supplement: S4 Fig — (DOCX) [file pone.0268903.s006.docx]

**Supplementary Fig. 4.** Funnel plot to detect publication bias in studies showing the serprevalence of *N. caninum* infection in the bovine aborted fetuses.
